# SFlt-1 Elevates Blood Pressure by Augmenting Endothelin-1-Mediated Vasoconstriction in Mice

**DOI:** 10.1371/journal.pone.0091897

**Published:** 2014-03-14

**Authors:** Fouad Amraoui, Léon Spijkers, Hajar Hassani Lahsinoui, Liffert Vogt, Joris van der Post, Stephan Peters, Gijs Afink, Carrie Ris-Stalpers, Bert-Jan van den Born

**Affiliations:** 1 Department of Internal and Vascular Medicine, Academic Medical Center, Amsterdam, The Netherlands; 2 Women’s and Children’s Clinic, Academic Medical Center, Amsterdam, The Netherlands; 3 Reproductive Biology Laboratory, Academic Medical Center, Amsterdam, The Netherlands; University of Southampton, United Kingdom

## Abstract

**Objective:**

Scavenging of vascular endothelial growth factor (VEGF) elevates blood pressure (BP) in patients receiving anti-angiogenic therapy. Similarly, inhibition of circulation VEGF by its soluble receptor fms-like tyrosine kinase-1 (sFlt-1) underlies BP elevation in pre-eclampsia. Both phenotypes are characterized by augmented production of endothelin-1 (ET-1), suggesting a role for ET-1 in anti-angiogenic hypertension. We aimed to assess the effect of VEGF inhibition on ET-1-induced contractility and downstream ET-1 signaling.

**Approach and Results:**

Male C57BL/6N mice were treated with either sFlt-1 or vehicle and BP was assessed via tail-cuff. Mean arterial pressure of sFlt-1-treated mice markedly increased compared to vehicle-treated controls (N = 11–12, *p*<0.05). After sacrifice, carotid and mesenteric arteries were isolated for isometric tension measurements. ET-1-induced contractions were similar in mesenteric arteries of vehicle and sFlt-1-treated mice, but augmented in carotid segments of sFlt-1-treated mice compared to controls (N = 9–10, *p*<0.05). The increased contraction in carotid segments could be completely abrogated by the cyclooxygenase (COX) inhibitor indomethacin (N = 9–10, *p*<0.05), indicating heightened prostaglandin-mediated vasoconstriction. This was associated with a shift towards procontractile ET_B_ signaling in sFlt-1-treated mice, possibly explaining the increased ET-1-induced prostaglandin-mediated vasoconstriction. In line with the *ex vivo* findings, sFlt-1-induced BP elevation could be prevented *in vivo* by oral treatment with either a high-dose of the COX inhibitor aspirin (N = 7) or with picotamide (N = 9), a dual thromboxane A_2_ synthase inhibitor and receptor antagonist.

**Conclusions:**

VEGF inhibition augments the pressor response to ET-1. The cyclooxygenase-thromboxane signaling route downstream of ET-1 might be a possible target to prevent BP elevation during VEGF inhibition.

## Introduction

Inhibition of vascular endothelial growth factor (VEGF) is causally related to the development of hypertension and proteinuria both in patients receiving anti-angiogenic therapy and in patients suffering from pre-eclampsia. In pre-eclampsia, circulating anti-angiogenic factors originating from the placenta contribute to the development of this pregnancy-related hypertensive disorder.[1]–[3] One of these anti-angiogenic factors is the soluble VEGF receptor fms-like tyrosine kinase-1 (sFLT-1) that scavenges circulating VEGF. sFLT-1 is expressed 3-fold higher in pre-eclamptic placentas compared to placentas from normotensive pregnancies and plasma sFLT-1 increases with the severity of pre-eclampsia.[3], [4] Elevated plasma sFLT-1 levels can be detected several weeks before onset of symptoms and rapidly decline after delivery.[3], [5].

When infused in pregnant and non-pregnant animals, sFlt-1 elicits hypertension, proteinuria and glomerular endotheliosis, which constitutes the histopathological hallmark of pre-eclampsia.[6], [7] Similarly, targeting VEGF directly with monoclonal antibodies or indirectly via tyrosine kinase inhibitors results in a pre-eclampsia-like syndrome, characterized by hypertension and proteinuria in humans and in animal models.[2], [8], [9].

VEGF stimulates nitric oxide (NO) production.[10] Inhibition of VEGF is therefore thought to decrease NO availability, thereby resulting in blood pressure (BP) elevation.[11] In addition, elevated levels of the potent vasoconstrictor endothelin-1 (ET-1) and its precursor preproendothelin have been detected in some, but not all, pre-eclamptic women and in plasma of patients treated with the tyrosine-kinase inhibitor Sunitinib.[8],[12],[13] Moreover, the rise in BP induced by VEGF inhibition can be fully reversed in animals by ET-1 receptor blockade with either a selective ET_A_ antagonist (ABT-627) as well as with a dual ET_A_ and ET_B_ antagonist (ACT-064992).[14], [15] However, additional contractile mechanisms, predominantly elevated prostanoid signaling, may exacerbate the ET-1-induced BP elevation during VEGF inhibition as ET-1 stimulates production of the vasocontractile prostanoid thromboxane A_2_ (TXA_2_).[16], [17] Production of TXA_2_ is indeed elevated in pre-eclampsia and results in a decreased prostacyclin/TXA_2_ ratio [18], [19].

In the present study, we aimed to assess whether VEGF inhibition with sFlt-1 increases the contractility towards ET-1. To this end, we treated C57/BL6N mice with either sFlt-1 or vehicle and carried out tail-cuff BP measurements. After sacrifice, we isolated carotid and mesenteric arteries for isometric tension measurements in a wire myograph. Infusion of sFlt-1 resulted in marked BP elevation and augmented ET-1 induced vasoconstriction in carotid artery segments but not in mesenteric segments. The increased contraction in carotid segments could be completely abrogated by the cyclooxygenase (COX) inhibitor indomethacin, indicating heightened ET-induced prostaglandin-mediated vasoconstriction. Accordingly, the sFlt-1-induced rise in BP could be prevented *in vivo* by oral treatment with the COX inhibitor aspirin and with picotamide, a dual TXA_2_ synthase inhibitor and receptor antagonist.

## Materials and Methods

### Animals and treatments

All experimental procedures in this study were approved by the Animal Ethics Committee of the Academic Medical Center, Amsterdam, The Netherlands (Permit Number: DFC102298). Adult 12-14 weeks old male C57/BL6N mice were purchased from Charles River and individually housed in a temperature controlled room with a 12:12 light-dark cycle and food and water *ad libitum*. After two weeks of acclimatization, the mice were anesthetized with isoflurane (2–4%) and osmotic minipumps (Alzet) were implanted subcutaneously. A single dose of buprenorphine (0.05 mg/kg *s.c.,* Schering-Plough) was administered for postoperative analgesia. The osmotic minipumps were filled with either vehicle (phosphate-buffered saline, PBS) or recombinant mouse sFlt-1 (Creative Biomart, catalog no: Flt1-1785M) for continuous 0.5μl/h compound release (equals 500 ng/h sFlt-1) during 2 weeks. Aspirin (30 mg/kg/day Cayman Chemical) or picotamide (5 mg/kg/day, Sigma) dissolved in minimal amounts of EtOH (<0.1%) were added to the drinking water during sFlt-1 treatment in a subset of mice.

During treatment, BP was recorded at fixed time intervals. After 2 weeks of treatment, the mice were euthanized by exsanguination during pentobarbital (O.P.G. Pharma) anesthesia (75 mg/kg *i.p*.) and carotid arteries were isolated for isometric analysis of vasomotor tone. Blood plasma (citrate), urine, kidneys and aortas were isolated, snap frozen in liquid nitrogen and stored at –80°C for further analysis.

### Blood pressure measurements

Non-invasive tail-cuff BP measurements were carried out in conscious mice using the CODA^tm^ system (Kent Scientific). To reduce BP variations in response to stress, BP measurements were carried out daily for one week just prior to the start of the experiment. The average of the first 15 subsequent BP measurement cycles was used to represent BP on each measurement day. BP measurements were performed between 3:00 – 5:00 pm.

Wire-myograph analyses. Carotid and mesenteric arteries were isolated and immediately placed in Krebs-Henseleit buffer (pH 7.4; in mmol/L: 118.5 NaCl, 4.7 KCl, 25.0 NaHCO_3_, 1.2 MgSO_4_, 1.8 CaCl_2_, 1.1 KH_2_PO_4_ and 5.6 glucose) for connective tissue removal and, in a small subset, endothelium-denudation. Artery segments of 2 mm were mounted into a multichannel wire myograph for isometric tension measurements as previously described.[20] In all experiments the segments were first contracted with high K^+^-containing Krebs-Henseleit buffer (100 mmol/L). After 30 minutes washout a concentration-response curve (CRC) of the α1-adrenoceptor agonist phenylephrine (Sigma) was generated with half-log concentration increments (1 nmol/L – 0.1 μmol/L). Contraction to phenylephrine was immediately followed by a methacholine (Sigma) CRC (1 nmol/L– 1 μmol/L) to assess endothelium-dependent vasodilatation. Next, after 15 minutes of washout, a high K^+^ Krebs-induced CRC (5 mmol/L – 100 mmol/L) was generated. Finally, after washout, artery segments were treated with either vehicle (5 μL DMSO, Merck), indomethacin (10 μmol/l, Sigma), L-Nitro Arginine Methyl Ester (L-NAME, 100 μmol/L, Sigma) and the selective ET_B_ receptor antagonist BQ788 (1 μmol/L, Bachem). An ET-1 (Bachem) CRC (0.1 nmol/L – 0.3 μmol/L) was generated in half-log concentration increments after 30 minutes incubation with each of the latter compounds and with endothelium-denuded carotid segments.

### Quantitative Real-Time PCR and tissue preparation

mRNA expression of genes encoding endothelin receptor type A (Ednra) and type B (Ednrb) was analyzed in mouse thoracic aorta by Quantitative Real-Time PCR (qPCR). RNA was isolated using MagNA Pure LC RNA Isolation Kit High Performance (Roche), and reverse transcribed using AMV First Strand cDNA Synthesis Kit for reverse transcription (RT)-PCR (Roche). qPCR was performed on a LightCycler 480 system (Roche) according to the manufacturer’s protocol with reaction mixtures containing 2.5 μl cDNA, 0.4 μmol/L of each primer (Life Technologies), 100 nmol/L UPL probe (Roche) and 10 μl Absolute qPCR mix (Thermo Scientific) in a total volume of 20 μl. Primer (shown in 5′ → 3′orientation)/probes were designed using the Roche Universal Probe Library Assay Design Center:

mEdnra GGGCATCACCGTCTTGAA/GGAAGCCACTGCTCTGTACC, probe UPL#99, mEdnrb TCAGAAAACAGCCTTCATGC/GCGGCAAGCAGAAGTAGAA, probe UPL#83, mHprt TGATAGATCCATTCCTATGACTGTAGA/AAGACATTCTTTCCAGTTAAAGTTGAG, probe UPL#22. QPCR data were analyzed and quantified using the second derivative maximum for Cp determination, with the LightCycler 480 software 1.5.0 (Roche).

### Analysis of renal morphology and proteinuria

Urine was collected at baseline and after treatment with either vehicle (PBS) or sFlt-1 to assess differences in proteinuria. Spot urine samples were collected by holding the mice over a sterile Petri dish. Total urinary protein concentration was assessed by a Pierce Coomassie Protein Assay kit (Thermo Scientific) according to the manufacturer’s instructions and is presented in absorbance units (AU). To assess the occurrence and severity of glomerular endotheliosis, we isolated kidneys of sFlt-1 and vehicle-treated mice. Periodic Acid-Schiff staining was applied on formalin-fixed and paraffin-embedded cross-sections for analysis. Kidneys were not flushed with formalin before fixation. From each kidney 50 micrographs were acquired at 40x magnification using the Olympus BX51 microscope equipped with an Olympus DP70 digital color camera (Olympus America). Glomeruli were selected randomly and blinded for prior treatment with vehicle or sFlt-1. Glomerular endotheliosis was then assessed by measurement of the glomerular open capillary volume, which was expressed as percentage of the total glomerular tuft area [7]. For this analysis we used publicly available ImageJ software [21].

### Statistical analyses

Contractile force of isolated arteries is presented in mN/mm and as percentage KCL contraction (%KCL) for graphic representation. Emax indicates the maximal contractile force and EC_50_ is defined as the concentration at which 50% of the maximal contraction is achieved. Concentration-response curves were fitted by non-linear regression analysis and EC_50_ was calculated using GraphPad Prism Software, CA, USA. Data are presented as means ± SEM, with ‘N’ being the number of individual mice. Changes from baseline in individual animals were assessed by paired student’s *t*-test. Between group differences were assessed by independent student’s *t*-test, one-way anova for more then two groups and two-way anova for assessing the effect of different treatments (sFlt-1 or vehicle) on Emax and EC_50_. All statistical analyses were performed using GraphPad Prism Software. Values of *p*<0.05 were considered to be statistically significant.

## Results

**Figure 1:**
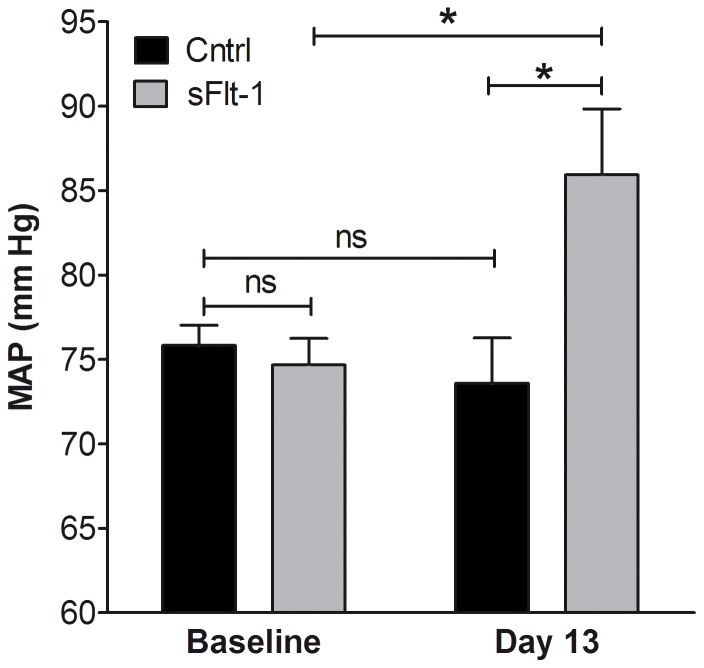
Effect of sFlt-1 on blood pressure. *In vivo* effect of sFlt-1 or vehicle (Cntrl) infusion during two weeks on mean arterial pressure (MAP). Data are presented as mean±SEM, N = 11–12, (ns) not significant, * *p*<0.05.

**Figure 2:**
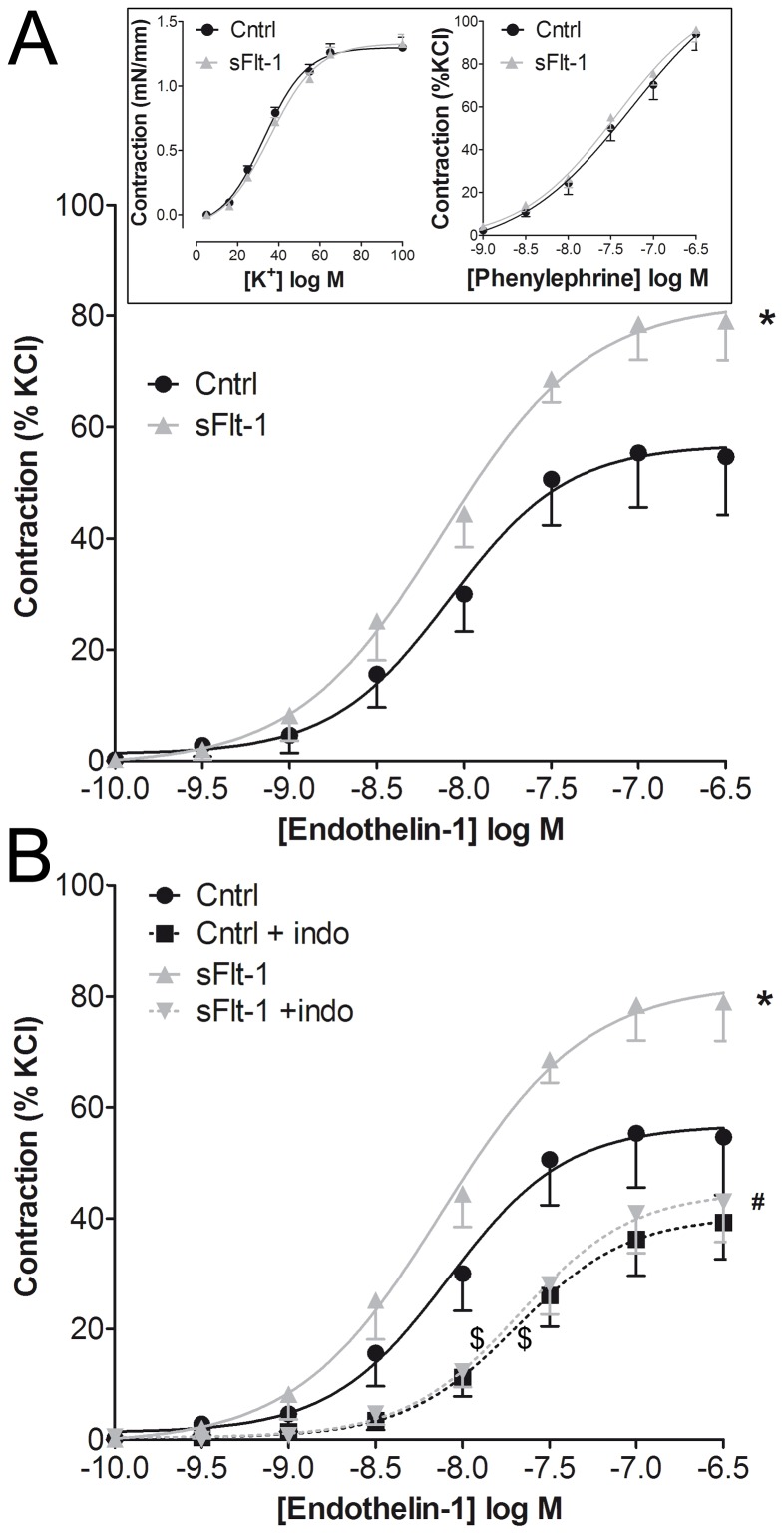
Effect of COX inhibition on endothelin-1 concentration-response curves. A) Concentration-response curve of endothelin-1 in isolated carotid arteries of vehicle-treated (Cntrl) and sFlt-1-treated mice. The inset depicts concentration-response curves of KCl (N = 15) and the α1-adrenergic receptor agonist phenylephrine (N = 8–9). **B**) Effect of pre-incubation with the non-selective cyclooxygenase-inhibitor indomethacin (indo; 10 μmol/L) on endothelin-1 concentration-response curves. Data are expressed as mean±SEM, *(maximal efficacy of sFlt-1 vs. Cntrl, N = 9–10, *p*<0.05), #(maximal efficacy of sFlt-1 vs. sFlt-1 + indo, N = 9–10, *p*<0.05), $(EC_50_ of sFlt-1 vs. sFlt-1 + indo and Cntrl vs. Cntrl + indo, *p*<0.05)

sFlt-1 infusion by osmotic minipump for a period of 2 weeks resulted in a marked elevation of BP compared to vehicle infusion (Figure 1). To assess whether increased ET-1-induced vasoconstriction contributed to this BP elevation, carotid and mesenteric arteries of vehicle-treated and sFlt-1-treated mice were isolated and mounted into a wire myograph. The maximal contraction towards ET-1 was increased in carotid segments of sFlt-1-treated mice (1.2±0.2 mN/mm, N = 10) compared to carotid segments of vehicle-treated mice (0.7±0.1 mN/mm, N = 9, (*p*<0.05, Figure 2A). There was no difference in the maximal ET-1-induced contraction between mesenteric arteries isolated from sFlt-1-treated mice (2.9±0.3 mN/mm, N = 6)) and vehicle-treated mice (3.0±0.4 mN/mm, N = 6, *p = *0.90). Additional wire myograph experiments were therefore carried out in carotid segments.

Pre-incubation with the non-selective COX inhibitor indomethacin abrogated the augmented maximal contraction of carotid segments in response to ET-1 in sFlt-1- treated mice, but not in vehicle-treated mice (Figure 2B), suggesting a role for contractile prostanoids in the augmented ET-1-induced contraction. The potency (EC_50_) of ET-1 induced contraction was significantly reduced by indomethacin in both vehicle and sFlt-1 treated mice (Figure 2B). Treatment with sFlt-1 did not affect potassium or phenylephrine contractions compared to vehicle treatment, suggesting that the increased contractility was specific for ET-1 (inset Figure 2A).

**Figure 3:**
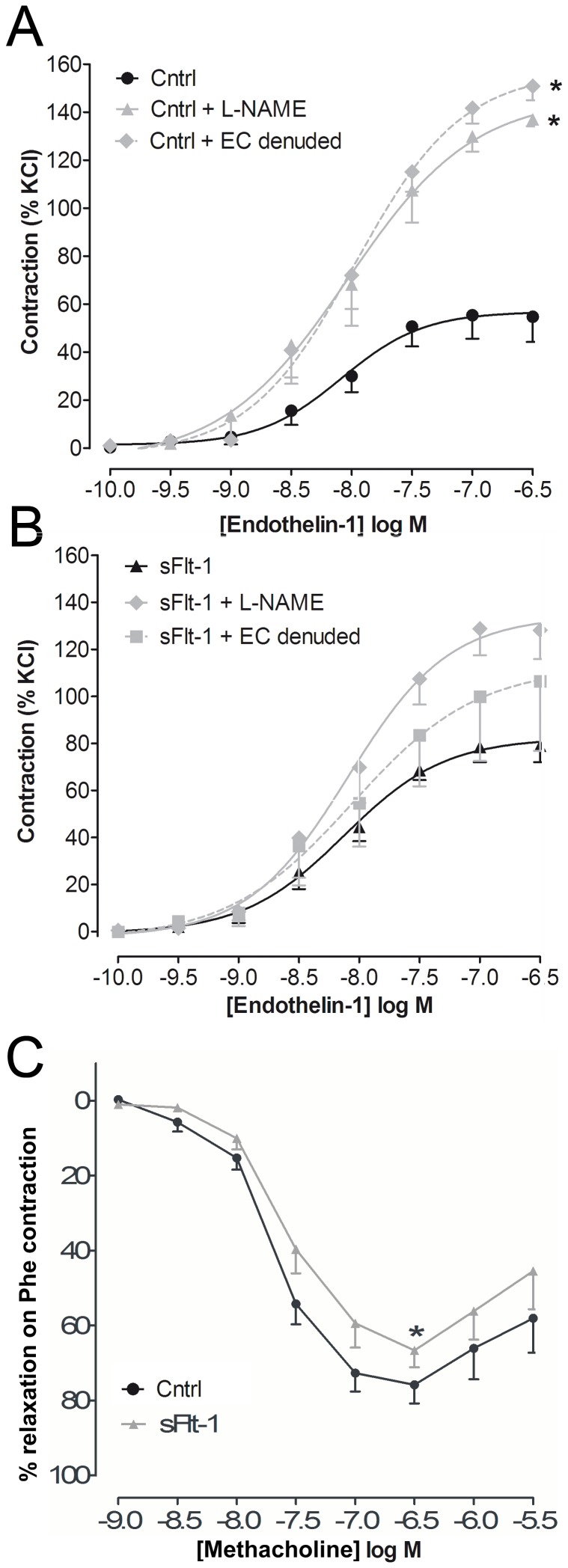
Role of nitric oxide and the endothelium. A) Effect of eNOS inhibition with L-NAME (N = 5) and endothelium (EC) denudation (N = 4) on ET-1 concentration-response curves in carotid segments of vehicle-treated mice (Cntrl). Data are expressed as mean±SEM, *(maximal efficacy of Cntrl vs. Cntrl + L-NAME and Cntrl vs. Cntrl + EC denuded, N = 9–5, *p*<0.05). **B**) Effect of L-NAME (N = 4) and EC denudation (N = 3) on ET-1 concentration-response curves in carotid segments of sFlt-1-treated mice. **C**) Metacholine concentration-response curve generated after pre-constriction with the α1-adrenergic receptor agonist phenylephrine. Data are expressed as mean±SEM,*(maximal relaxation sFlt-1 vs. Cntrl, N = 13–14, *p*<0.05).

To assess whether the increased ET-1-induced contraction was endothelium dependent, carotid segments of sFlt-1-treated mice were either endothelium-denuded or incubated with the endothelial nitric oxide synthase (eNOS) inhibitor L-NAME. Endothelium-denudation increased contraction to ET-1 from 0.7±0.1 mN/mm to 1.0±0.1 mN/mm (*p*<0.01) in carotid segments of vehicle-treated mice, but did not significantly increase contraction in segments of sFlt-1-treated mice (*p* = 0.10, Figure 3A+B). Furthermore, inhibition of eNOS by L-NAME increased ET-1-induced contraction from 0.7±0.1 mN/mm to 1.3±0.2 mN/mm (*p*<0.01) in segments of vehicle-treated mice (*p*<0.01), but not in sFlt-1-treated mice (*p* = 0.44, Figure 3A+B) indicating decreased residual NO availability in sFlt-1-treated mice. In agreement with this differential effect of eNOS inhibition, the maximal relaxation potential of carotid segments to methacholine during phenylephrine contraction was significantly lower in carotid arteries of sFlt-1-treated mice compared to vehicle-treated mice (*p*<0.05, Figure 3C).

**Figure 4:**
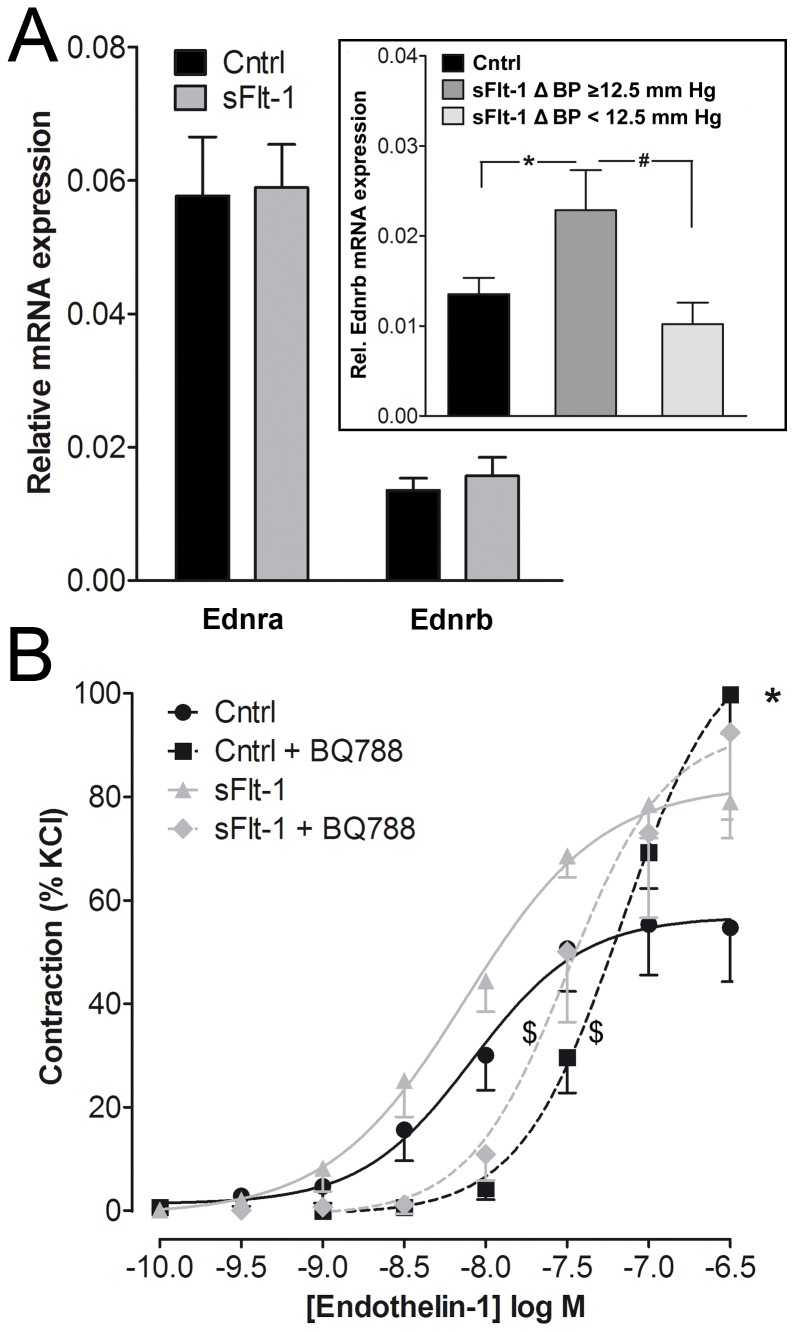
mRNA expression of ET_A_ and ET_B_ receptors and effect ET_B_ receptor blockade. A) Quantitative Real-Time PCR showing thoracic aorta mRNA expression profiles of Ednra and Ednrb in vehicle (Cntrl) and sFlt-1-treated mice normalized to Hprt mRNA levels. Data expressed as mean±SEM, *(Inset figure shows a two-fold increase in high-responders vs. Cntrl, N =  7–13, *p*<0.05), #(high-responders vs. low-responders, N = 7–5, *p* = 0.06). **B**) Effect of pre-incubation with the ET_B_ receptor antagonist BQ788 on endothelin-1 concentration-response curves of carotid segments isolated from vehicle (Cntrl, N = 5) and sFlt-1-treated mice (N = 4). *(maximal efficacy of Cntrl vs. Cntrl + BQ788, N = 5–9, *p*<0.05), $(EC_50_ of sFlt-1 vs. sFlt-1 + BQ788 and Cntrl vs. Cntrl + BQ788, *p*<0.05)

To investigate whether mRNA expression of the ET-1 receptors (Ednra and Ednrb) was altered, we evaluated thoracic aorta receptor mRNA presence via real-time PCR. Expression of Ednra and Ednrb was not altered in the thoracic aorta of sFlt-1-treated mice compared to vehicle-treated mice (Figure 4A). However, Ednrb expression in sFlt-1-treated mice with a high BP increase according to median split, was elevated compared to vehicle-treated mice (*p*<0.05, inset Figure 4A). Ednra expression was not differentially expressed in sFlt-1-treated mice with a high or mild BP increase. Incubation with the ET_B_ receptor antagonist BQ788 (1 μmol/L) before generation of ET-1 concentration response curves elevated the EC_50_ in both sFlt-1 as well as in vehicle-treated mice (Figure 4B). Still, BQ788 augmented maximal ET-1-induced contraction in segments of vehicle-treated mice from 0.7±0.1 mN/mm to 1.1±0.2 mN/mm (*p*<0.01) but not in segments of sFlt-1-treated mice (Figure 4B, *p* = 0.41), suggesting that sFlt-1 altered ET_B_ receptor signaling.

**Figure 5:**
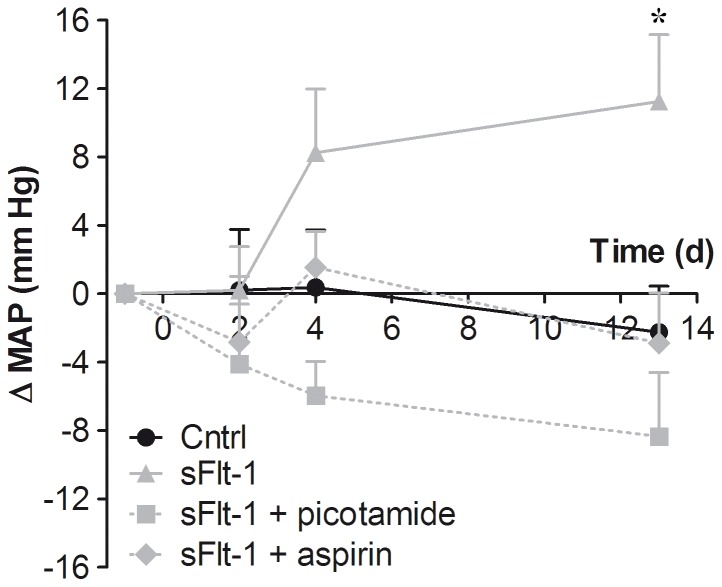
Effect of aspirin and picotamide on sFlt-1-induced BP elevation. The *in vivo* effect of intervention with aspirin (30 mg/kg/day) or picotamide (5 mg/kg/day) during two weeks on mean arterial pressure (MAP). Data are expressed as mean±SEM, N = 7–12. *(sFlt-1 vs Cntrl, *p*<0.05 with one-way anova and Dunnets post-hoc test.

Next, we evaluated the *in vivo* implications of the apparent increase in ET-1 induced prostanoid-mediated vasoconstriction in sFlt-1-treated mice. We assessed whether the beneficial effects of prostanoid inhibition, as presented in isolated artery segments, could be validated to prevent BP increases as well. Aspirin (30 mg/kg/day) and picotamide (5 mg/kg/day), a dual TXA_2_ synthase inhibitor and thromboxane prostanoid (TP) receptor antagonist, were administered concurrently to sFlt-1 treatment. Baseline mean arterial pressure (MAP) of aspirin treated mice was 80±2 mmHg and picotamide-treated mice had a baseline MAP of 84±3 mmHg. Both aspirin and picotamide markedly prevented the rise in BP induced by sFlt-1 (Figure 5). MAP of aspirin-treated mice displayed a tracing similar to that of vehicle-treated mice with no significant change after 13 days compared to baseline. MAP of picotamide-treated mice tended to decrease but this was also not significantly different compared to baseline and vehicle-treated mice.

Finally, urinalysis at baseline showed similar urinary protein excretion in sFlt-1 (N = 6, 0.32±0.02 AU) and vehicle-treated mice (N = 7, 0.36±0.02 AU, *p* = 0.29) and this did not change from baseline after two weeks of treatment with vehicle (0.37±0.02 AU, *p* = 0.99) and sFlt-1 (0.33±0.02 AU, *p* = 0.57). The percentage open glomerular capillary volume, as indicator of endotheliosis, was also not different between vehicle (53±1%, N = 5) and sFlt-1-treated mice (52±2%, N = 5, *p = *0.66).

## Discussion

In the present study we show that sFlt-1 infusion in mice augments ET-1-mediated vasoconstriction, thereby contributing to BP elevation. COX-inhibition prevented the increased ET-1-induced vasosconstriction *ex vivo* and rise in BP *in vivo*, indicating that ET-1 promotes signaling of contractile prostanoids after sFlt-1 treatment.

Increased circulating levels of ET-1 in women with pre-eclampsia was already reported two decades ago,[22], [23] but has only recently been associated with VEGF inhibition by sFlt-1.[12] VEGF inhibition in animals and treatment of humans with the tyrosine kinase inhibitor sunitinib, have shown to result in elevated renal cortex mRNA expression of preproendothelin and increased circulating peptide levels of ET-1.[8], [14] Here, we show that ET-1-induced vasoconstriction is enhanced by VEGF inhibition. This augmented contraction observed after VEGF inhibition may be partially explained by a decreased NO bioavailability, as VEGF stimulates NO production by eNOS.[10], [11], [24] The increased circulating ET-1 associated with VEGF inhibition may result from this decrease in NO availability as NO attenuates ET-1 synthesis.[25] By inhibiting eNOS with L-NAME during ET-1 concentration-response curves, we demonstrated a significantly increased contraction in vascular segments of vehicle-treated mice, but not in segments of sFlt-1-treated mice, indicating decreased residual NO availability in sFlt-1-treated mice. Decreased NO availability was also suggested by the decreased NO-mediated vasorelaxation in response to metacholine in sFlt-1-treated mice.

The role of ET-1 in mediating the increase in BP associated with VEGF inhibition is supported by the previous observations that ET_A_ receptor antagonism prevents the rise in BP and that VEGF inhibition with sFlt-1 increases ET_A_ mRNA expression in the renal cortex of female C57/BL6 mice.[7], [8], [14] Despite significant BP elevation, we did not observe any change in ET_A_ mRNA expression in the thoracic aorta, suggesting differential effect of sFlt-1 on ET_A_ mRNA expression in various tissues. We assessed ET-1-induced vasoconstriction in carotid arteries, which might have differential ET_A_ expression compared to the thoracic aorta. In fact, ET_A_ mRNA expression may even be decreased in placental and myometrial tissue of women with pre-eclampsia compared to healthy pregnant women.[26] Interestingly, myometrial mRNA expression of the ET_B_ receptor was increased in women with pre-eclampsia. This is in line with our observation of increased ET_B_ expression in thoracic aortas of sFlt-1-treated mice with a large BP increase. The functional relevance of altered ET_B_ signaling is displayed by the discriminate effects of the selective ET_B_ antagonist BQ778 between vehicle and sFlt-1 conditions. BQ778 augmented the maximal contractility to ET-1 in vehicle-treated mice but not in sFlt-1-treated mice was, suggesting that sFlt-1 might have reduced the ability of endothelium-situated ET_B_ receptors to induce vasodilation. The importance of altered endothelial signaling was further demonstrated by the increased contractility towards ET-1 in endothelium-denuded vascular segments of vehicle-treated mice, which could not be achieved in endothelium-denuded segments of sFlt-1-treated mice. Together these observations may suggest a shift towards predominantly contractile signaling for endothelial ET_B,_ which has previously been shown to mediate release of the contractile prostanoid TXA_2_ by ET-1.[27] Besides augmenting the maximal ET-1-induced contraction in vehicle-treated mice, BQ788 elevated EC_50_ in both groups equally. This inhibitory effect of BQ788 might be caused by its weak ET_A_ antagonistic properties.[27]

COX inhibition with indomethacin reduced the potency of ET-1 in both vehicle and sFlt-1-treated mice. However, only in carotid arteries of sFlt-1-treated mice was the augmented maximal efficacy of ET-1 completely prevented by indomethacin. This indicates that sFlt-1 treatment increased ET-induced prostaglandin-mediated vascoconstriction. Since e*x vivo* activation of the TP receptor by TXA_2_ induces similar contractions in sFlt-1 and vehicle-treated mice,[28] it seems unlikely that expression of the TP receptor is altered by sFlt-1 treatment. The relevance of elevated prostanoid signaling induced by sFlt-1 is demonstrated by *in vivo* treatment with aspirin and picotamide, which both prevented the rise in BP induced by sFlt-1.

Several previous studies have shown a consistent rise in BP after sFlt-1 treatment in mice using invasive as well as non-invasive techniques for the measurement of BP. The median increase in MAP induced by sFlt-1 infusion in the current study is comparable to the BP increase reported in non-pregnant female C57/BL6 mice during andenovirus-mediated overexpression of sFlt-1, but slightly lower compared to viral overexpression of sFlt-1 in pregnant BALB/c and CD1 mice.[7], [29], [30] Of note is that we used a tail-cuff which tends to slightly underestimate BP compared to telemetry.[31] Delivery of sFlt-1 via an osmotic minipump has not been previously reported for mice, but has been shown to increase BP in pregnant rats.[6], [14].

The increased ET-1-induced contraction in carotid segments was not observed in mesenteric arteries which, in general, contribute more to peripheral resistance and BP elevation. This discrepancy may not be related to isolation and mounting of these smaller vessels, since KCL, phenylephrine and metacholine curves were generated prior to ET-1 constriction curves and showed no evidence for endothelial or smooth muscle injury. Interestingly, ET-1induced vasoconstriction is also not augmented in mesenteric arteries of pregnant rats with surgically reduced uteroplacental perfusion pressure compared with sham-operated rats.[32] This well established rat model for pre-eclampsia is also associated with increased sFlt-1 levels.[33] A differential contractile response of conduit and resistance arteries has been reported previously in other experimental hypertensive phenotypes and might be due to the fact that conduit arteries are more sensitive to decreases in NO availability.[34] Besides increased peripheral resistance, diminished NO availability and increased ET-1-induced vasocontrsiction in the kidney may lead to changes in renal hemodynamics and contribute to long-term BP elevation associated with VEGF inhibition.[35] We observed no significant increase in total urinary protein level and no glomerular endotheliosis after sFlt-1 treatment. It is unlikely that the absence of kidney injury might be attributed to the moderate increase in BP, as similar elevations of BP have previously been shown to coincide with proteinuria in C57BL/6 mice.[7], [9] Glomerular changes and proteinuria may, however, depend on the achieved plasma sFlt-1 concentration.[29] Duration of exposure to peak plasma sFlt-1 levels might be longer with adenoviral-mediated overexpression compared to infusion with osmotic minipumps. Nevertheless, the risk for developing hypertension also exceeds that of developing proteinuria in patients treated with different doses of the VEGF-antibody bevacizumab.[2], [36]In conclusion, the present results demonstrate that sFlt-1 augments ET-1 induced vasoconstriction, thereby contributing to BP elevation in mice. Inhibition of the prostanoid signaling pathway abrogated the increased vasoconstriction to ET-1 *ex vivo* and prevented the sFlt-1-induced rise in BP *in vivo*. This suggests that sFlt-1 increases ET-1-induced release of contractile prostanoids, which exacerbate ET-1-induced vasoconstriction and thus contribute to BP elevation. Aspirin reduces the risk of cardiovascular events in patients receiving anti-VEGF therapy with bevacizumab,[37] but its potential role in preventing BP elevation has not been addressed previously. Whether the beneficial effect of aspirin in the prevention of pre-eclampsia may indeed be extrapolated onto the pre-eclampsia-like syndrome needs to be confirmed in future clinical studies.[38] The benefit of preventing BP elevation in these patients may lie in reducing the risk of hypertensive crises and subsequent cessation of life-prolonging anti-angiogenic treatment rather than in cardiovascular risk management.[39].
